# Resection of distal gastric tube cancer with sentinel node biopsy: a case report and review of the literature

**DOI:** 10.1186/s12957-014-0421-5

**Published:** 2015-01-28

**Authors:** Yasumichi Yagi, Toru Ii, Shigehiro Tanaka, Hikaru Oguri

**Affiliations:** Department of Surgery, Koseiren Namerikawa Hospital, 119 Tokiwa-cho, Namerikawa, 936-8585 Japan; Department of Surgery, Toyama City Hospital, 2-1 Imaizumi Hokubu-machi, Toyama, 939-8511 Japan; Department of Internal Medicine, Koseiren Namerikawa Hospital, 119 Tokiwa-cho, Namerikawa, 936-8585 Japan

**Keywords:** Gastric tube cancer, Distal resection, Sentinel node biopsy

## Abstract

**Background:**

The frequency of gastric tube cancershas increased with advances in surgical techniques and improvement of survival rates in patients with esophageal cancer. However, a standard surgical treatment has not yet been established. Total resection of the gastric tube with lymphadenectomy has been considered a radical treatment, while repeat surgery with both laparotomy and thoracotomy has been associated with severe complications, including anastomotic leakage, recurrent nerve paralysis, bronchotracheal injury, and damage to other organs.

**Case presentation:**

We present a successful case of a gastric tube cancer that was treated with surgical resection in combination with sentinel node biopsy. The tumor was diagnosed as a type 0-IIc lesion with ulceration, and was located proximal to the pyloric ring. Endoscopic submucosal dissection was not indicated because the primary lesion was submucosally invasive, and undifferentiated. By the dye-guided method, sentinel nodes were detected along the right gastroepiploic artery and vein. Intraoperative pathological examination revealed no metastasis of the sentinel nodes. Resection of the distal gastric tube was safely performed with a Roux-en-Y reconstruction, preserving the right gastroepiploic artery and vein and the perfusion of the proximal gastric tube.

**Conclusion:**

We suggest distal resection of the gastric tube with sentinel node biopsy as a novel surgical method for a cT1N0 gastric tube cancer located in the abdomen.

## Background

Gastric tube cancers (GTCs) are gastric cancers that arise in reconstructed gastric tubes (GTs). Their frequency has increased with advances in surgical techniques and improvement of survival rates in esophageal cancer patients, occurring in around 1.7 to 8.6% of patients [1,2]. Previously, most cases of GTCs presented at an advanced stage, and therefore had a poor prognosis [3]. However, periodic endoscopic examination has contributed to improvement in prognosis, enabling us to detect early-stage GTC, and allowing the use of less invasive treatments, such as endoscopic submucosal dissection (ESD). A standard surgical strategy for GTC has not yet been established. Total resection of the GT with lymphadenectomy has been considered a radical treatment. Repeat surgery with both laparotomy and thoracotomy is associated with severe complications, including leakage of the anastomosis, recurrent nerve paralysis, bronchotracheal injury, and damage to other organs. Moreover, open operative procedures seem to be excessively invasive for early-stage GTCs. One less invasive option that has been employed is open palliative surgery without lymphadenectomy, which involves partial excision and wedge resection of the GT; unfortunately, this is insufficient in terms of treating any lymph node metastasis. In addition, repairing GT defect after wedge resection with sufficient surgical margin is difficult and complicated; suturing in the direction of the long axis may lead to stenosis of the GT, and suturing in the direction of the short axis may fail to repair with tension of the GT. Partial resection of the GT without lymphadenectomy would be reasonable if the absence of nodal metastasis could be confirmed and blood flow to the remnant GT could be maintained.

In gastric cancer, surgical treatment has become increasingly less invasive after the development of sentinel node (SN) navigation surgery [4,5]. A SN is defined as the first node drained from the primary lesion and has been proven to be a reliable indicator of regional lymph node metastasis in a variety of solid tumors [6,7]; a negative SN therefore implies the absence of regional lymph node metastasis. SN biopsy has been successfully used in gastric cancers as an intraoperative diagnostic method to determine the extent of lymph node dissection [8,9]. Similarly, SN navigation could have a role to play in any potentially minimally invasive treatment of GTCs.

The surgical management of GTC should be based on that of gastric cancer because of the many similarities they share in vascularity and lymphatic route. Obvious differences include the removed portions of the left gastric artery, the left gastroepiploic artery, and the posterior gastric artery. Because the blood supply to a reconstructed GT after esophagectomy occurs mainly through the right gastroepiploic artery (RGEA) and the right gastric artery (RGA), progression of GTC via the lymphatic tract would be simple. Thereafter, under the limited lymphatic route of GTs, SN navigation surgery can be applied equally for cases of GTC according to gastric cancer treatment. We therefore present a successful case of GTC treated by minimally invasive surgery with resection of the distal GT in combination with SN navigation. To the best of our knowledge, the present case is the first report describing the surgical treatment of GTC using SN navigation.

## Case presentation

**Figure 1:**
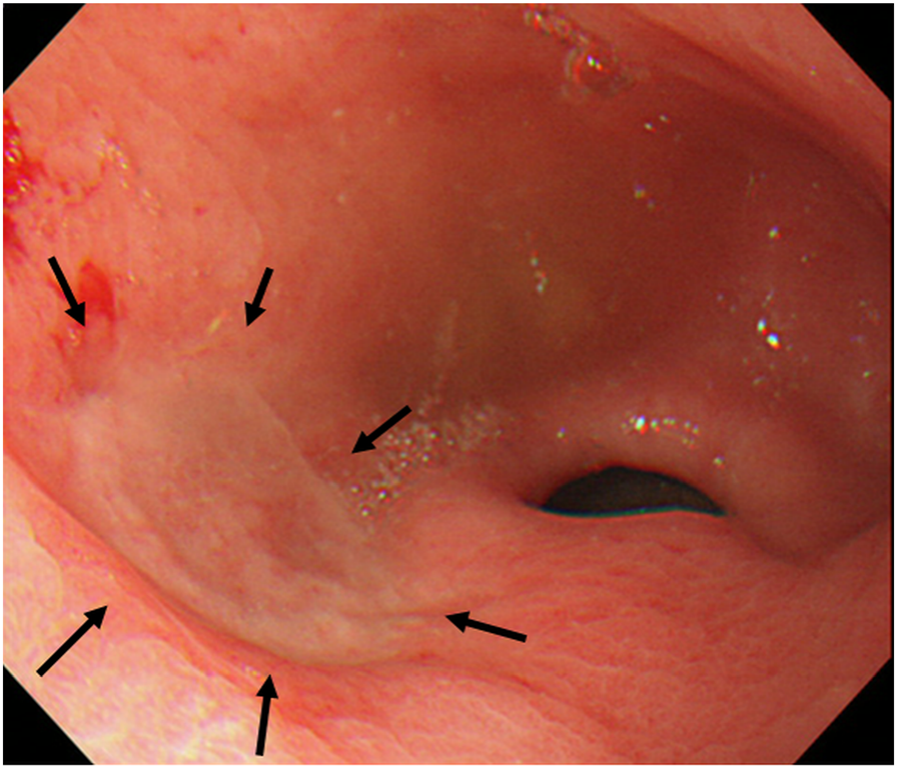
**Endoscopic findings.** A type 0-IIc tumor was located on the oral side of the pyloric ring (arrows) and the depth of the lesion was estimated to be submucosal.

**Figure 2:**
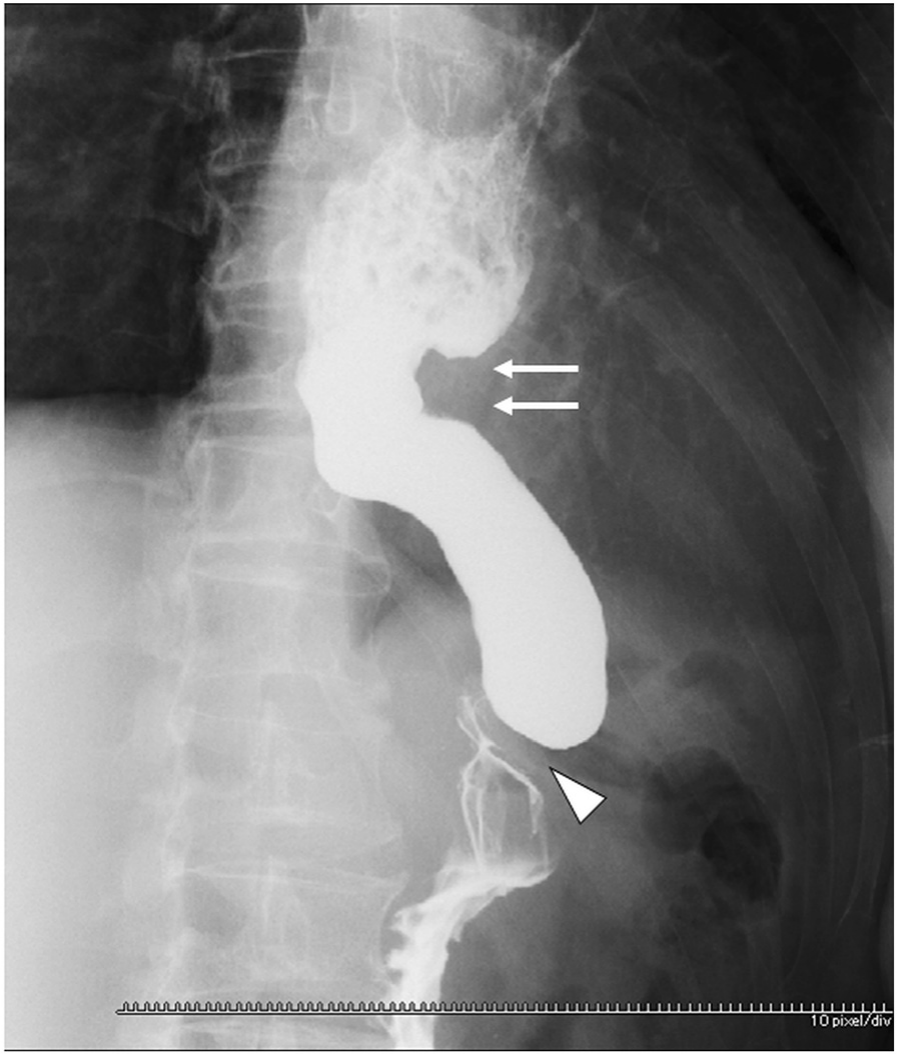
**Upper gastrography.** The abdominal gastric tube was about 5 cm from the pyloric ring (white arrows) to the thoraco-abdominal border (white arrowhead). The primary lesion was not detected.

A 72-year-old man had undergone subtotal esophagectomy with GT reconstruction via the posterior mediastinal route for thoracic esophageal cancer. Pathological diagnosis of the resected esophageal cancer revealed a poorly differentiated squamous cell carcinoma with invasion of the adventitia that was diagnosed as pT3N1M0, Stage III. He had an otherwise uneventful recovery; however, a GTC was found during routine follow-up 4.5 years after the original subtotal esophagectomy. Endoscopic examination showed a type 0-II c tumor with ulceration, located proximal to the pyloric ring (Figure 1). Biopsies were taken, and the histological examination ultimately led to a diagnosis of signet-ring cell carcinoma. Barium swallow was unable to detect the primary tumor, although the shape of the GT exhibited narrowing at the border of the thorax and abdomen (Figure 2). Computed tomography was also unable to detect the primary tumor and found no obvious nodal metastasis. The preoperative stage of the GTC was therefore diagnosed as cT1N0M0, Stage I. On the basis of guidelines for treating gastric cancer, ESD was not indicated for the GTC because the lesion was undifferentiated and estimated to be submucosal in depth. All other laboratory values, including tumor markers, were within normal limits. The therapeutic strategy was explained to the patient, who opted for undergoing surgical intervention.

### Sentinel node biopsy for gastric tube cancer

**Figure 3:**
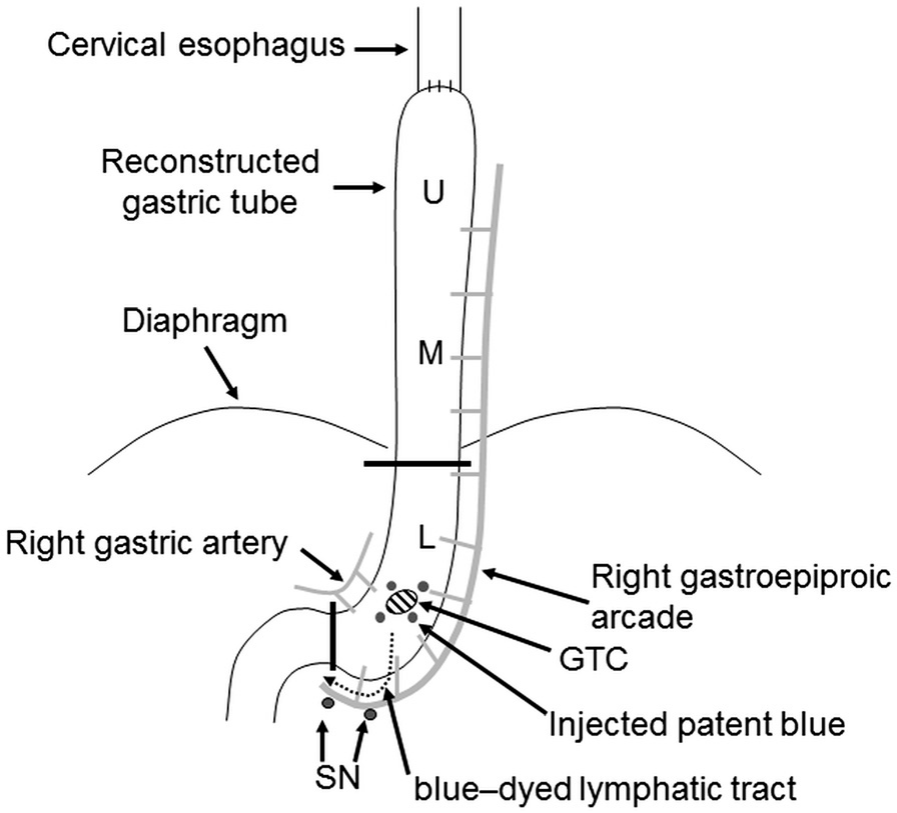
**Diagrammatic representation for resection of the distal gastric tube in combination with sentinel node biopsy.** After injection of patent blue, the blue-dyed lymphatic tract was visualized from the primary tumor to only the right gastroepiploic artery and two blue nodes were detected. GTC, gastric tube cancer; L, lower gastric tube; M, middle gastric tube; SN, sentinel node; U, upper gastric tube.

The remaining gastrocolic ligament was divided to visualize the direction of lymphatic flow from the stomach. Under intraoperative endoscopy, 2% patent blue was injected into the submucosal layer at four quadrants around the primary lesion using an endoscopic puncture needle. After approximately 10 minutes, we could visualize the blue-dyed lymphatic tract from the primary tumor to only the RGEA, but we could not detect the upward lymphatic route in the mediastinum. At the edge of the upper stream of the lymphatic tract, two blue nodes were detected, excised, and transferred to the department of pathology for intraoperative diagnosis. These blue-dyed lymph nodes were defined as SNs (Figure 3). While awaiting the pathological results, both the RGEA and right gastroepiploic vein (RGEV) were carefully delineated. Frozen sections of the SNs revealed no evidence of metastasis. Consequently, there was no need for lymphadenectomy, and the perfusion of the proximal GT could be maintained by preserving the gastroepiploic arcade.

### Distal resection of the gastric tube and Roux-en-Y reconstruction

**Figure 4:**
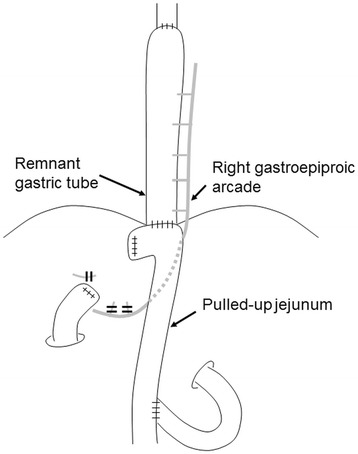
**Diagrammatic representation for Roux-en-Y reconstruction.** Gastroepiploic arcade was preserved with maintenance of the upper gastric tube perfusion after distal resection of the gastric tube.

**Figure 5:**
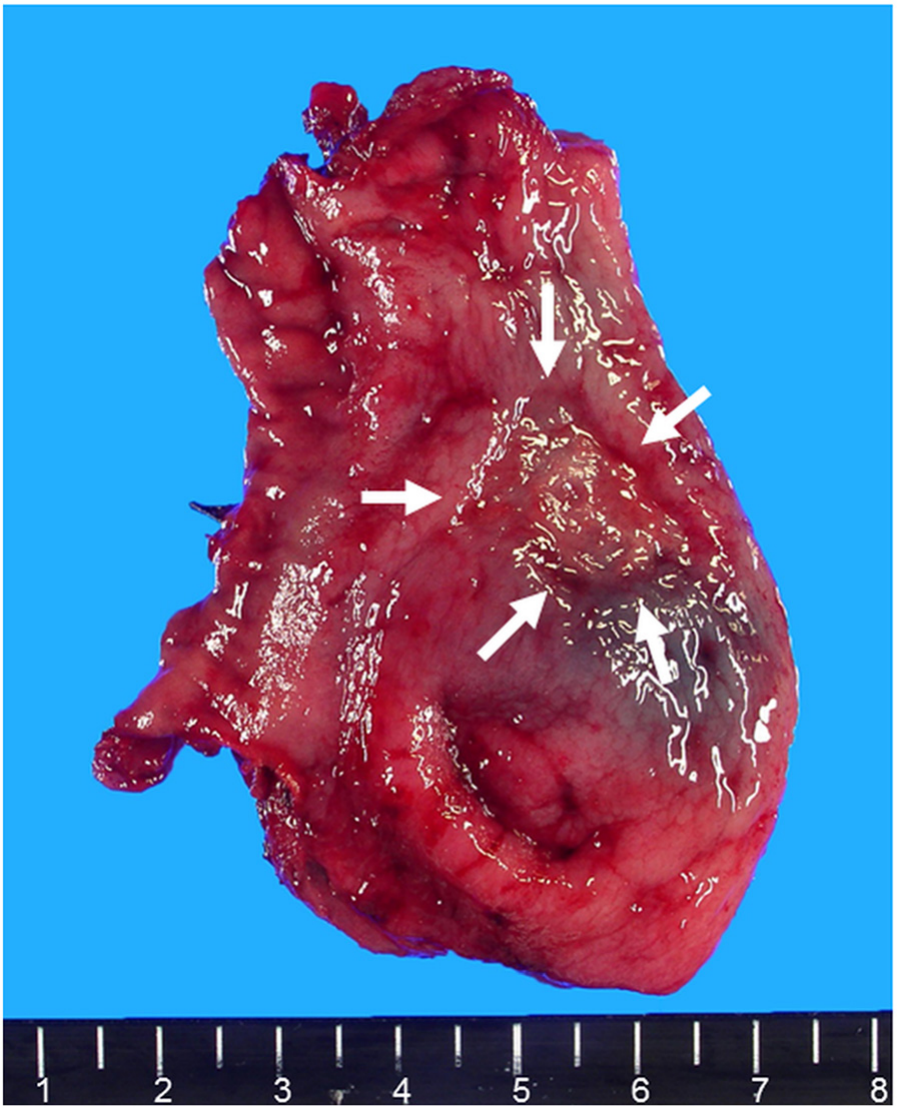
**Resected gastric tube cancer specimen.** At the pre-pylorus, a type 0-IIc tumor presented with sufficient surgical margins (white arrows). The proximal and distal resection margins were 10 and 22 mm, respectively.

After the SN biopsy, the duodenum was cut off using a GIA60 stapler (Covidien, Mansfield, MA, USA) with lymphadenectomy of the RGA area. The GT was resected at the border between the thorax and abdomen using purse-string sutures (Figure 3). The surgeon inserted an anvil into the stump of the GT, and a gastrojejunostomy was performed with a circular stapler (EEA; Covidien), and a Roux-en Y reconstruction was completed (Figure 4). Thus, resection of the distal GT was successfully carried out, preserving the thoracic GT without the need for a thoracotomy. Sufficient surgical margins were confirmed on the resected specimens (Figure 5). The postoperative period was uneventful.

Pathological examination of the resected specimen revealed a signet-ring cell carcinoma with submucosal invasion of more than 500 μm, without both lymphatic and venous infiltration, diagnosed as pT1N0M0, Stage I. No evidence of recurrence has been found after 24 months of follow-up.

## Discussion

Lymph node metastasis is one of the most important prognostic factors in patients with gastric cancer [10-12]. Radical gastrectomy with extended (D2) removal of regional lymph nodes is the standard treatment for curable gastric cancer, and it has improved the survival of patients with nodal metastasis [13,14]. Conversely, prophylactic lymphadenectomy provides low efficacy in patients with probability of negative nodal involvement. Lymph node metastasis with submucosal invasion occurs in 15 to 20% of gastric cancer patients [15], meaning that lymphadenectomy may be unnecessary for the remaining 80 to 85% of patients. Regardless of this inconsistency, a given proportion of patients with clinically node-negative cancers undergo routine D2 lymphadenectomy as a standard intervention [9]. If we can predict the node status in these patients, it may be possible to offer minimally invasive surgery with personalized lymphadenectomy.

The frequency of nodal metastasis in GTC has been reported according to the depth of tumor invasion as follows: 0% in intramucosal tumors, 10% in submucosal tumors, 28% in tumors with muscularis propria invasion, 46.7% in subserosal tumors, and 54.5% in serosal tumors [2]. A probable reason for the low incidence of lymph node metastasis in early GTC is that early cancer metastasis via the lymphatic route may be prevented in the devascularized GT, as is the case in remnant gastric cancer [16]. Where nodal metastasis is not clinically present, ESD would be a good option for intramucosal or submucosal GTC. The accepted indications for curative resection using ESD in cT1 gastric cancer are intramucosal and differentiated tumors, tumors <2 cm in diameter, and the absence of ulceration [17]. The 2010 Japanese Gastric Cancer Association guidelines for the treatment of submucosal gastric cancer specify the following as extended criteria for curative endoscopic resection: size ≤30 mm; differentiated-dominant histology; lack of vessel involvement; and submucosal invasion of <500 μm [17]. According to these gastric cancer treatments, surgical resection of the GTC in combination with SN biopsy would be a reasonable procedure for a lesion of undifferentiated type or with risk of nodal metastasis. To our knowledge, the present case is the first report of SN navigation surgery applied to the management of GTC.

In gastric cancer, Miwa and colleagues employed the dye mapping technique to identify the SNs of gastric cancer for the first time [18], and reported a high positive predictive value and accuracy for SN biopsy in the early stages [19]. Thus, SN biopsy has facilitated the implementation of less invasive surgery, associated with the preservation of the stomach and the reduction in the need for the extent of lymphadenectomy. Clinically, a cT1N0 lesion has been considered a good indication for SN navigation surgery [20]. On the other hand, a T3 tumor is considered a contraindication for the same because of the high probability of nodal metastasis, and obstruction of the lymphatic drainage routes affects the ability to detect SNs. Therefore, even in cT2N0 GTC, a negative SN would reliably indicate the absence of nodal metastases with a high degree of accuracy.

The main methods used in SN navigation surgery are node-pickup biopsy and lymphatic basin dissection. Basin dissection is lymphadenectomy focused to the region that contains SNs. The lymphatic basins, defined as the area containing the stained lymphatic vessel, are able to be divided into the following five categories according to the direction of arterial flow surrounding the stomach: the left gastric artery area, the RGA area, the left gastroepiploic artery area, the RGEA area, and the posterior gastric artery area. In the present case, we employed pickup biopsy using the dye-guided method to preserve the RGEA and RGEV. Since cT1 gastric cancer is not always palpable from the serosal aspect, and accurate injection is technically difficult by the subserosal approach [20], we employed endoscopic guidance for accurate dye injection. Osaka and colleagues described that SNs detected by the dye-guided method is a reliable approach [21]. In addition, the dye-guided method enables us to detect lymphatic flow and gives us more advantage to clarify the region of lymphadenectomy in surgical treatment of GTC. To improve accuracy and decrease false negativity in SN detection, if possible, the double tracer method would be preferable using a combination of dye and radioisotope tracers [22]; the false negative rate was 4.3% using the dye-guided method alone [23].

The GT, otherwise referred to as pulled-up stomach, is the most common esophageal substitute selected for reconstruction after esophagectomy. To describe the location of the GTC, we divided the GT into three regions: the upper GT (the upper half of the thoracic GT including the esophagogastric anastomosis), the middle GT (the lower half of the thoracic GT), and the lower GT (the distal or abdominal GT). Blood flow to the GT after esophagectomy is principally supplied by the RGEA and RGA as with the RGEV for drainage. Additionally, collateral circulation develops over time following reconstruction of the GT, with intramural communication of blood flow through the anastomosis. Saito and colleagues [24] described that revascularization might occur in the proximal region of the GT through the anastomosis, from the cervical esophagus, in a patient surviving several years after esophagectomy. Liebermann-Meffert and colleagues [25] demonstrated that the upper 20% of the GT is only perfused through microcirculation. Following GT reconstruction, a collateral vascular network develops between the upper GT and the cervical esophagus and predominates over the right gastroepiploic arcade. Accordingly, the blood flow of any given GT is dependent upon the site of the given GT: the upper GT depends on intramural perfusion via the anastomosis; the middle GT is supplied by the RGEA and drained by the RGEV; and the lower GT is supplied mainly by the RGEA and drained by the RGEV with intramural perfusion from the duodenum. Although this alternation of perfusion in the upper GT also might imply an altered lymphatic flow around the esophagogastrostomy, there have been no reports in the literature of skip metastases in upper GTCs beyond the esophagogastric anastomosis.

The extent of GT resection should therefore be decided according to the blood flow to the GT. Saito and colleagues [24] reported two cases of subtotal resection of the GT with resection of the RGEA and preservation of the upper GT. In these patients, the blood supply to the GT was evaluated by indocyanine green fluorescence imaging and confirmed as passing from the remnant esophagus to the upper GT through the esophagogastric anastomosis. As the blood flow was confirmed up to about 5 cm from the anastomotic line, they successfully preserved about 3 cm of the upper region with a 2 cm safety margin. Thus, the proximal (upper) GT depends exclusively on intramural perfusion from the anastomosis, and resection does not have to be limited to the anastomosis or preservation of the RGEA for subtotal resection of the GT. For middle GTC, subtotal resection of the GT with lymphadenectomy would be recommended. Accordingly, the resection of the RGEA and RGEV would not significantly influence the blood flow of the remnant GT. For upper GTC, total resection of the GT would need to be considered. For lower GTC, resection of the abdominal GT would be sufficient. However, the RGEA and RGEV must be preserved, since blood may not be supplied by intramural perfusion from the cervical esophagus.

Lymphadenectomy carried out with resection of the distal GT requires ligation of the RGEA, leading to ischemia of the lower and middle GT. Yoshida and colleagues [26] reported two cases of distal GT resection with dissection of the RGEA and RGEV that necessitated vascular reconstruction. They estimated blood flow to the remnant GT by macroscopic appearance, such as color and bleeding. In our case, SN biopsy enabled us to avoid lymphadenectomy and to perform resection of the distal GT and preserve the RGEA. In SN-negative GTC, resection of the distal GT can be safely performed with preservation of the RGEA and RGEV. In SN-positive lower GTC, resection of the distal GT and resection of the RGEA and RGEV with vascular reconstruction can be employed. In lower GTC, SN navigation surgery has the possibility to reduce the need for a thoracotomy by limiting lymphadenectomy and facilitating minimal resection of the GT with preservation of the RGEA and RGEV. Due to prolonged post-esophagectomy survival rate, GTC is no longer a rare disease and is set to become more prevalent in the future. Thereafter, our surgical method with SN biopsy will contribute to broaden treatment options for GTC. Although SN navigation surgery is regarded as an available procedure in gastric cancer, further examination of this surgical procedure should be considered for its usefulness in GTC.

## Conclusion

Distal resection of the GT with SN biopsy is a novel surgical method and a treatment option for a cT1N0 GTC located in the abdomen. To our knowledge, the present case is the first report describing the surgical treatment of GTC using the SN concept.

## Consent

Written informed consent was obtained from the patient for publication of this case report and any accompanying images. A copy of the written consent is available for review by the Editor-in-Chief of this journal. Patient consent was obtained for the study presented in the manuscript.

## Abbreviations

ESD: endoscopic submucosal dissection
GT: gastric tube
GTC: gastric tube cancer
RGA: right gastric artery
RGEA: right gastroepiploic artery
RGEV: right gastroepiploic vein
SN: sentinel node
